# Isolated Tillaux Fracture in Adults – Literature Review Based on a Rare Case Report

**DOI:** 10.1055/s-0042-1743270

**Published:** 2022-03-11

**Authors:** Nuno Vieira da Silva, Ana Esteves, Pedro Ribeiro, José Miradouro, Joana Pereira, Julio Marinheiro

**Affiliations:** 1 Departamento de Ortopedia e Traumatologia, Centro Hospitalar do Tâmega e Sousa, Penafiel, Portugal

**Keywords:** ankle fractures, ankle injuries, bone screws, tibial fractures

## Abstract

Isolated Tillaux fracture is a rare anterolateral distal tibia fracture frequently misdiagnosed in adults. It typically occurs in adolescents nearing skeletal maturity by avulsion of the anterior-inferior tibiofibular ligament. This case-based literature review study aims to elicit the existing information regarding this fracture in adults, and summarize its injury mechanism, diagnosis, and treatment procedures. According to the literature, this is only the eighth case described: a 46-year-old woman that suffered an isolated Tillaux fracture with 4 mm of displacement, and open reduction and fixation with double cannulated screws were performed. After proper rehabilitation, an excellent functional and radiological outcome was reached. It is important to recognize and appropriately treat these distinct injuries to prevent further instability, degenerative changes, and ankle joint function limitation. Early diagnosis and appropriate osteosynthesis play a significant role in a successful recovery prognosis.

## Introduction

Tillaux fractures are avulsion fractures of the anterior-inferior tibiofibular ligament (AITFL) from its tibial attachment, which typically occur in adolescents nearing skeletal maturity (12–14-years-old). This fracture pattern has not been commonly seen in an older population, since the ligaments usually fail before the bone. 1

The incidence of isolated Tillaux fractures in adults has not been quantified in the current data, as it has been more commonly seen in association with other injuries. According to the literature, only 7 case reports have been published regarding this entity. 2

The present case-report-based literature review aims to elicit the existing information regarding isolated Tillaux fractures in adults and provide a summary about its mechanism of injury, diagnosis, and treatment procedures.

## Case Report

A 46-year-old woman was admitted to the emergency department after a domestic ankle trauma with external rotation mechanism. Physical examination revealed tenderness and swelling over the anterolateral aspect of the ankle with normal but painful passive range of motion.

**Fig. 1:**
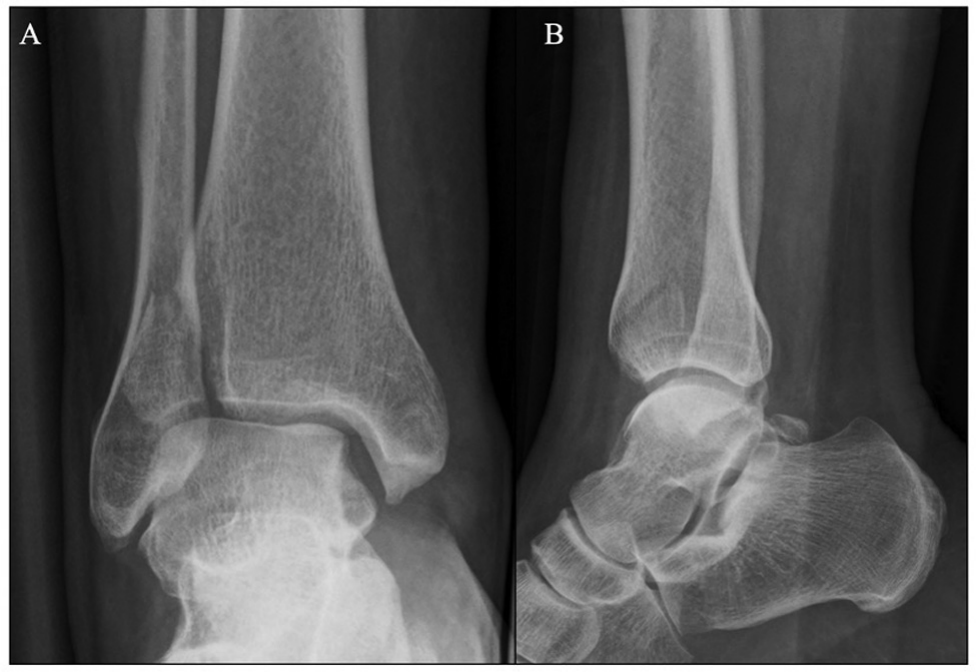
Preoperative X-ray view: ( **A** ) anteroposterior and ( **B** ) lateral.

**Fig. 2:**
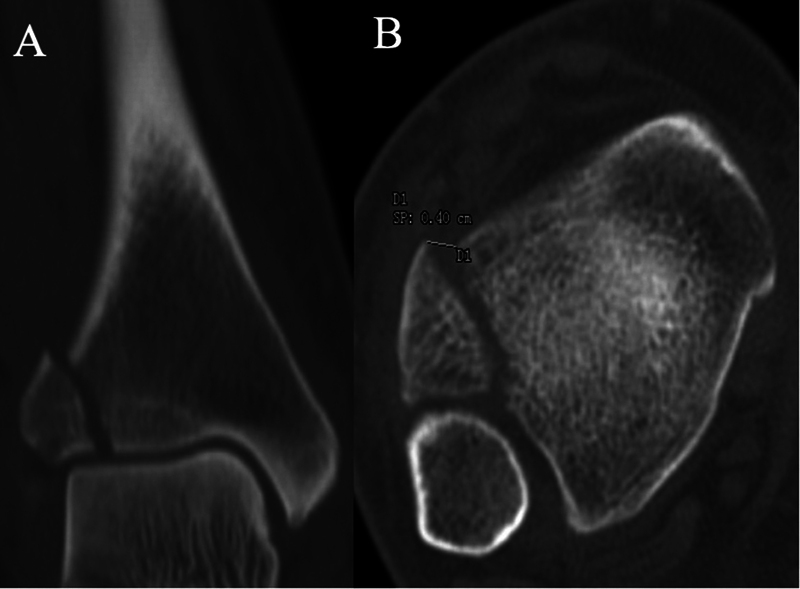
Preoperative CT scan view: ( **A** ) coronal and ( **B** ) axial.

The images of x-ray ( Fig. 1 ) and computed tomography (CT) scan ( Fig. 2 ) showed an isolated Tillaux fracture with 4 mm of fragments displacement and anterior disruption of the syndesmosis. Open reduction and fixation were performed with 2 cannulated 4.0 screws, perpendicular to the fracture line by a mini-open anterolateral approach—proximally centered between the tibia and fibula, and distally extended in line with the fourth metatarsal in an intermuscular plane between the peroneus brevis and tertius. The superficial peroneal nerve runs anterior to the fibula and requires identification in the proximal extent of the incision. 3

**Fig. 3:**
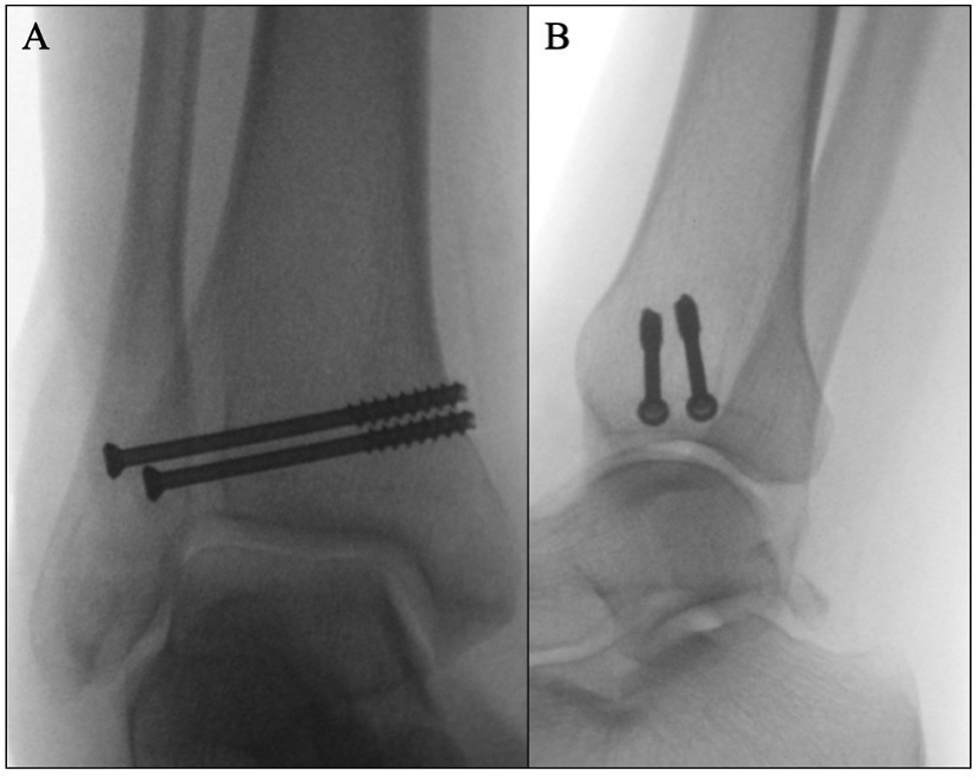
Intraoperative fluoroscopic imaging view: ( **A** ) anteroposterior and ( **B** ) lateral.

Fluoroscopic imaging ( Fig. 3 ) was used intraoperatively to confirm fracture reduction, correct positioning of the screws, and syndesmotic stability with the negative external rotation stress test.

Postoperatively, a short leg cast was prescribed during 4 weeks for fear of patient's lack of compliance with the no weight-bearing discharge instructions. Mobilization started at the 4 ^th^ week, partial weight-bearing was allowed from the 6 ^th^ week onwards, and was gradually increased according to clinical and radiological evidence of union up to full weight-bearing and normal walking.

**Fig. 4:**
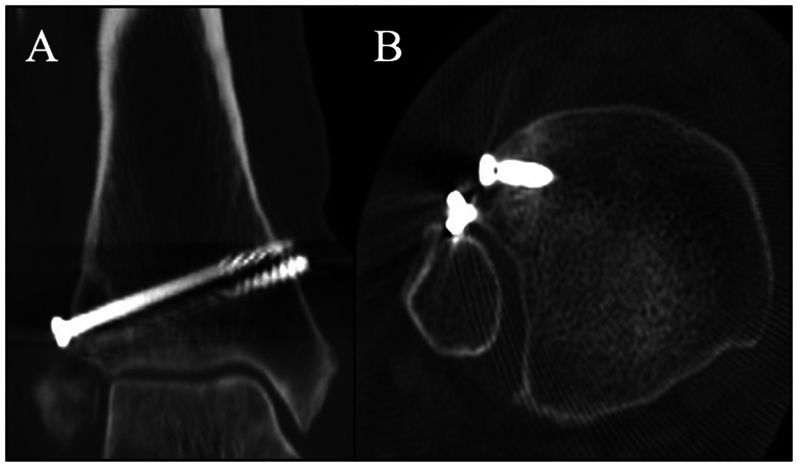
Postoperative control CT scan view: ( **A** ) coronal and ( **B** ) axial.

After 4 months of proper rehabilitation, the patient exhibited an excellent clinical and functional outcome, scoring 80 in the Karlsson scale. A postoperative control CT scan ( Fig. 4 ) confirmed a successful bone healing.

## Discussion

Historically, this fracture pattern has been noted in the adolescent population due to the pattern of progression of physeal closure and classified as a Salter-Harris III fracture through the epiphysis. 4

Ankle syndesmosis is formed by the distal tibia and fibula, and it is stabilized by four ligaments: the anterior, transverse, and posterior tibiofibular ligaments, as well as the interosseous membrane. 2

Supination and external rotation (SER) were identified as the most common mechanism of injury; leading to avulsion of the anterolateral tibia site of attachment of the anterior inferior tibiofibular ligament. This mechanism of injury is typically divided into 4 stages. However, in isolated Tillaux fracture scenarios, the sequence of events ends at stage 1, since no fibula fracture, posterior osseous injury, or medial involvement are present. 1 Fall from height is also described as a possible injury mechanism. 2

Nondisplaced Tillaux fractures are often almost unrecognizable on standard X-ray projections and may be misdiagnosed as a simple sprain due to its challenging diagnosis. Stress X-ray imaging and oblique projection should be used as supplemental diagnostictools. 1 Additional CT evaluation is also recommended for determining the distance of displacement, fracture fragments shape, and conditions of the articular surface. 4

In adult Tillaux fractures the avulsed fragment is generally triangular, while in juvenile Tillaux fractures it is quadrangular. 5

A total of three main types of fractures can be differentiated: (1) extra-articular avulsion fracture of the AITFL, (2) fracture of the anterolateral distal tibia with involvement of the articular surface, and (3) impaction fracture of the anterolateral tibial plafond. 6

Nondisplaced fractures (< 2 mm) with no evidence of syndesmosis instability can be managed conservatively by long leg cast with internally rotated foot. Since the strong anteroinferior ligament is attached to the fibula, it renders the bone displaced and angulated, causing syndesmotic incompetency. Therefore, a displaced fracture (> 2mm) is an indication for close or open reduction and internal fixation. 7

Conservative treatment of dislocated fragments leads to non-union and post-traumatic osteoarthritis. Impaction fractures can lead to secondary avascular necrosis of the anterolateral tibial plafond. 6

The aim of surgical fixation of displaced anterolateral distal tibial fractures is the anatomical stabilization of the anterior syndesmosis and restoration of the tibial incisura for the distal fibula and joint surface. A type 1 fracture may be fixed with an anchor or transosseous suture, type 2 is mostly fixed with screws, and type 3 may need bone grafting of the impaction zone for restoration of the joint surface and buttress plating. 6

It is not yet clear whether the fibula should be temporarily fixed to the tibia but, irrespective of the type of treatment, fixation for six weeks with instructions not to load the injured limb is indicated. 2

Open reduction can be done by anterior or anterolateral approach—it should depend on the extent of the fracture line. Using the anterolateral approach, a second approach to address a medial component is often necessary in more severe injuries. On the other hand, using the anterior approach, the entire anterior portion of the distal tibia is accessible, but the neurovascular bundle is at higher risk both proximally and distally. 3

Percutaneous fixation techniques have also been described to treat this injury, predominantly among adolescents. 8 Arthroscopically assisted fixation technique has been described but no actual literature evidence suggests that this method is overall superior to traditional open reduction. 9 It represents a more accurate method with treatment possibility of any associated intra-articular pathology and lower risk of infection, bleeding, and biological damage to structures. 1

As a mainly intra-articular fracture, anatomical reduction, absolute stability, and early mobilization are the most important factors to ensure a better functional outcome. 10

Fraturas isoladas de Tillaux em adultos podem ser indicativas de uma lesão sindesmótica no tornozelo. É importante reconhecer e tratar adequadamente essas lesões distintas para evitar maiores instabilidades, alterações degenerativas e limitação da função articular do tornozelo.

Além da rara ocorrência na idade adulta, outra característica dessa fratura é que ela é desafiadora para detectar, sendo facilmente negligenciada. Um diagnóstico precoce e a osteossíntese apropriada desempenham um papel significativo em um prognóstico de recuperação bem-sucedido.

Isolated Tillaux fractures in adults may be indicative of a syndesmotic ankle injury. It is important to recognize and appropriately treat these distinct injuries to prevent further instability, degenerative changes, and ankle joint function limitation.

In addition to the rare occurrence in adulthood, another feature of this fracture is that it is challenging to detect, being easily overlooked. An early diagnosis and appropriate osteosynthesis play a significant role in a successful recovery prognosis.

## References

[1] Kose O Yuksel H Y Guler F Ege T Isolated Adult Tillaux Fracture Associated With Volkmann Fracture-A Unique Combination of Injuries: Report of Two Cases and Review of the Literature J Foot Ankle Surg 2016 55 05 1057 1062 26711834 10.1053/j.jfas.2015.10.005

[2] Gasparova M Falougy H E Kubikova E Almasi J Isolated “Tillaux” fracture in adulthood: rarity where the key of success is not to miss it Bratisl Lek Listy 2020 121 08 533 536 32726113 10.4149/BLL_2020_088

[3] Hickerson L E Verbeek D O Klinger C E Helfet D L Anterolateral Approach to the Pilon J Orthop Trauma 2016 30 02 S39 S40 27441938 10.1097/BOT.0000000000000611

[4] Oak N R Sabb B J Kadakia A R Irwin T A Isolated adult Tillaux fracture: a report of two cases J Foot Ankle Surg 2014 53 04 489 492 24795204 10.1053/j.jfas.2014.03.012

[5] Sharma B Reddy I S Meanock C The adult Tillaux fracture: one not to miss BMJ Case Rep 2013 2013 bcr2013200105 10.1136/bcr-2013-200105 PMC3736651 23868026

[6] Rammelt S Bartoníček J Neumann A P Kroker L [Fractures of the anterolateral tibial rim : The fourth malleolus] Unfallchirurg 2021 124 03 212 221 33580301 10.1007/s00113-021-00959-y

[7] Mishra P K Patidar V Singh S P Chaput Tubercle Fracture in an Adult- A Rare Case Report J Clin Diagn Res 2017 11 03 RD01 RD02 10.7860/JCDR/2017/21567.9524 PMC5427393 28511467

[8] Feng S M Sun Q Q Wang A G Li C K “All-Inside” Arthroscopic Treatment of Tillaux-Chaput Fractures: Clinical Experience and Outcomes Analysis J Foot Ankle Surg 2018 57 01 56 59 29037924 10.1053/j.jfas.2017.07.020

[9] Kumar N Prasad M Tillaux fracture of the ankle in an adult: a rare injury J Foot Ankle Surg 2014 53 06 757 758 25128312 10.1053/j.jfas.2014.06.010

[10] Syed T Storey P Rocha R Kocheta A Singhai S Tillaux Fracture in Adult: A Case Report Ortho J MPC 2020 26 02 95 98. Available from:https://ojmpc.com/index.php/ojmpc/article/view/126

