# Supplementary material for: Isolated Tillaux Fracture in Adults – Literature Review Based on a Rare Case Report
Source: Rev Bras Ortop (Sao Paulo). 2022 Mar 11;59(Suppl 1):e52–5. doi: 10.1055/s-0042-1743270 (PMC11254419; doi:10.1055/s-0042-1743270)
Supplement: Related texts

# Research article: Fratura isolada de Tillaux em adulto – Revisão da literatura com base em um relato de caso raro

**Keywords:** fraturas da tíbia, fraturas do tornozelo, parafusos ósseos, traumatismos do tornozelo

## Abstract

A fratura isolada de Tillaux representa uma rara e frequentemente mal diagnosticada fratura de tíbia distal anterolateral em adultos. Normalmente ocorre em adolescentes que se aproximam da maturidade esquelética por avulsão do ligamento tibiofibular anteroinferior. Este estudo de revisão de literatura baseada em caso tem como objetivo lembrar a existência dessa identidade em adultos, resumir seu mecanismo de lesão, diagnóstico e procedimentos de tratamento. De acordo com a literatura, este é apenas o oitavo caso descrito: uma mulher de 46 anos sofreu uma fratura isolada de Tillaux com 4 mm de deslocamento e realizaram-se redução e fixação aberta com parafuso canulado duplo. Após a reabilitação adequada, alcançou-se um excelente desfecho funcional e radiológico. É importante reconhecer e tratar adequadamente essas lesões distintas para evitar maiores instabilidades, alterações degenerativas e limitação da função articular do tornozelo. Um diagnóstico precoce e a osteossíntese apropriada desempenham um papel significativo em um prognóstico de recuperação bem-sucedido.

## Introdução

As fraturas de Tillaux são fraturas de avulsão do ligamento tibiofibular anteroinferior (LTFAI) a partir de seu apego tibial e normalmente ocorrem em adolescentes que se aproximam da maturidade esquelética (12–14 anos). Este padrão de fratura não é visto comumente em uma população mais velha, uma vez que os ligamentos geralmente falham antes do osso. 1

A incidência de fraturas isoladas de Tillaux em adultos ainda não foi quantificada nos dados atuais, pois tem sido mais comumente observada em associação com outras lesões. De acordo com a literatura, apenas 7 relatos de casos foram publicados sobre essa entidade. 2

A presente revisão da literatura baseada em relatório de caso tem como objetivo relembrar as informações existentes sobre fraturas isoladas de Tillaux em adultos e fornecer um sumário sobre seu mecanismo de lesão, diagnóstico e tratamento.

## Relato de Caso

Uma mulher de 46 anos deu entrada no pronto-socorro após um trauma doméstico no tornozelo, com mecanismo de rotação externa. O exame físico revelou sensibilidade e inchaço sobre o aspecto anterolateral do tornozelo com amplitude passiva normal, mas dolorosa.

As imagens de raio X (Fig. 1 ) e tomografia computadorizada ( Fig. 2 ) mostraram uma fratura isolada de Tillaux com 4 mm de deslocamento de fragmentos e interrupção anterior da sindesmose. A redução e fixação abertas foram realizadas com 2 parafusos 4.0 cânulados, perpendiculares à linha de fratura por uma abordagem anterolateral mini-aberta – centrada proximalmente entre tíbia e fíbula. e estendida distalmente em linha com o quarto metatarso em um plano intermuscular entre peroneus brevis e tertius. O nervo peroneal superficial corre antes da fíbula e requer identificação na extensão proximal da incisão. 3

**Fig. 1:** Visão de raio X pré-operatório: ( **A** ) anteroposterior e ( **B** ) lateral.

**Fig. 2:** Visão da tomografia pré-operatória: ( **A** ) coronal e ( **B** ) axial.

A imagem fluoroscópica ( Fig. 3 ) foi utilizada intraoperatóriamente para confirmar a redução da fratura, o posicionamento correto dos parafusos e estabilidade sindesmótica com o teste de estresse de rotação externa negativo.

**Fig. 3:** Visão de imagem fluoroscópica intraoperatória: ( **A** ) anteroposterior e ( **B** ) lateral.

No pós-operatório, um gesso de perna curto foi prescrito durante 4 semanas por medo da falta de cumprimento das instruções em relação à descarga sem peso por parte da paciente. A mobilização foi autorizada a partir da 4ª semana e o rolamento parcial de peso foi permitido a partir da 6ª semana, aumentando gradualmente de acordo com evidências clínicas e radiológicas de consolidação, até a caminhada sob peso total.

Após 4 meses de reabilitação adequada, a paciente apresentou um excelente resultado clínico e funcional, marcando 80 na escala de Karlsson. A tomografia computadorizada de controle pós-operatório ( Fig. 4 ) confirmou uma cicatrização óssea bem-sucedida.

**Fig. 4:** Visão da tomografia computadorizada de controle pós-operatório: ( **A** ) coronal e ( **B** ) axial.

## Discussão

Historicamente, esse padrão de fratura tem sido observado na população adolescente devido ao padrão de progressão do fechamento fiseal e é classificado como uma fratura de Salter-Harris III através da epífise. 4

A sindesmose do tornozelo é formada pela tíbia distal e fíbula e é estabilizada por quatro ligamentos: os ligamentos tibiofibular anterior, transversal e posterior, e a membrana interossea. 2

A supinação e a rotação externa (SRE) foram identificadas como o mecanismo mais comum de lesão; levando à avulsão do local da tíbia anterolateral de fixação do ligamento tibiofibular inferior anterior. Este mecanismo de lesão é tipicamente dividido em 4 estágios. No entanto, em cenários isolados de fratura de Tillaux *,* a sequência de eventos termina no estágio 1, uma vez que não há fratura de fíbula, lesão óssea posterior ou envolvimento medial. 1 Quedas de altura também são descrita como um possível mecanismo de lesão. 2

As fraturas de Tillaux não deslocadas são muitas vezes quase irreconhecíveis nas projeções padrão de raios X e podem ser mal diagnosticadas como uma simples entorse devido ao seu diagnóstico desafiador. Imagens de raio X de estresse e a projeção oblíqua devem ser usados como ferramentas de diagnóstico suplementares. 1 Recomenda-se também uma avaliação adicional da tomografia computadorizada para determinar a quantidade de deslocamento, forma de fragmentos de fratura e condições da superfície articular. 4

Nas fraturas de Tillaux em adultos, o fragmento avulso é geralmente triangular, enquanto nas fraturas de Tillaux juvenis é quadrangular. 5

Um total de três tipos principais de fraturas podem ser diferenciados: (1) fratura de avulsão extra-articular do LTFAT, (2) fratura da tíbia distal anterolateral com envolvimento da superfície articular e (3) fratura de impacto do plafona tibial anterolateral. 6

Fraturas não deslocadas (< 2 mm) sem evidência de instabilidade de sindesmose podem ser administradas conservadoramente com gessos de perna longa com o pé girado internamente. Uma vez que o ligamento anteroinferior forte é ligado à fíbula, ele a torna deslocada e angulada, causando incompetência sindesmótica. Portanto, uma fratura deslocada (> 2 mm) é uma indicação para redução proximal ou aberta, e fixação interna. 7

O tratamento conservador de fragmentos deslocados leva à osteoartrite não sindicalizada e pós-traumática. Fraturas de impacto podem levar à necrose avascular secundária do plafonado tibial anterolateral. 6

A fixação cirúrgica de fraturas tibiais distal anterolateral deslocadas visa a estabilização anatômica da sindesmose anterior, restauração da incisura tibial para a fíbula distal e a superfície articular. Fraturas de tipo 1 podem ser fixadas com uma sutura âncora ou transóssea, as tipo 2 são fixadas principalmente com parafusos, e as tipo 3 podem precisar de enxerto ósseo da zona de impacto para restauração da superfície articular e do revestimento da bunda. 6

Ainda não está claro se a fíbula deve ser temporariamente fixada na tíbia, mas, independentemente do tipo de tratamento, a fixação por seis semanas sem permissão para colocar peso sobre o membro machucado é indicada. 2

A redução aberta pode ser feita por abordagem anterior ou anterolateral – deve depender da extensão da linha de fratura. Usando a abordagem anterolateral, uma segunda abordagem para abordar um componente medial é frequentemente necessária em lesões mais graves. Por outro lado, usando a abordagem anterior, toda a porção anterior da tíbia distal é acessível, mas o feixe neurovascular está em maior risco tanto proximal quanto distalmente. 3

Técnicas de fixação percutânea também foram descritas para tratar essa lesão, predominantemente entre adolescentes. 8

A técnica de fixação assistida artroscopicamente foi descrita, mas nenhuma evidência real da literatura sugere que este método é globalmente superior à redução aberta tradicional. 9 Representa um método mais preciso com possibilidade de tratamento de qualquer patologia intra-articular associada, e com um risco menor de infecção, sangramento e danos biológicos às estruturas. 1

Por ser uma fratura principalmente intra-articular, a redução anatômica, a estabilidade absoluta e a mobilização precoce são os fatores mais importantes para garantir um melhor resultado funcional. 10
